# Learning to Practice Compassionate Care: Medical Students Discuss Their Most Memorable Lessons

**DOI:** 10.1177/23743735221117383

**Published:** 2022-08-04

**Authors:** Cynthia E. Schairer, Jenna Tutjer, Christopher Cannavino, William C. Mobley, Lisa Eyler, Cinnamon S. Bloss

**Affiliations:** 1 T. Denny Sanford Institute for Empathy and Compassion, 8784 University of California San Diego, La Jolla, CA, USA; 2 Herbert Wertheim School of Public Health and Human Longevity Science, 8784 University of California San Diego, La Jolla, CA, USA; 3 Department of Pediatrics, School of Medicine, 8784 University of California San Diego, La Jolla, CA, USA; 4 Department of Neuroscience, School of Medicine, 8784 University of California San Diego, La Jolla, CA, USA; 5 Department of Psychiatry, School of Medicine, 8784 University of California San Diego, La Jolla, CA, USA

**Keywords:** compassionate care, undergraduate medical education, focus group research, navigation techniques, perspective taking

## Abstract

Compassion in interactions between physicians and patients can have a therapeutic effect independent of the technical medical treatment provided. However, training physicians to effectively communicate compassion is challenging. This study explores how medical students experienced training focused on interacting with patients by examining students’ reports of particularly memorable lessons. Six focus groups were conducted with medical students (total n = 48) in their fourth year of training. We report on responses from students to the question, “What was the most memorable lesson you have learned about interacting with patients?” Students discussed lessons aimed at patient-centered physical navigation, interpersonal navigation, and perspective taking. Concerns were raised that navigation techniques felt inauthentic and that perspective taking was too time consuming to be sustainable in actual practice. While perspective-taking exercises should motivate medical students to treat every patient with dignity by demonstrating the complexity of others’ lives, if students assume that full understanding is a prerequisite to delivery of compassionate care, they may dismiss explicit techniques of patient-centered care as inauthentic and perceive compassion and efficiency as mutually exclusive.

## Introduction

The importance of empathic and compassionate doctor–patient relationships has become more widely appreciated in recent years. There is growing evidence that empathic and compassionate interactions can have a therapeutic effect independent of the technical treatment provided (1–4). In addition to saving lives, compassionate care has been shown to save money and lessen provider burnout (4–6). Research has shown that compassionate communications that improve health can integrate into efficient high-quality treatment (4,7,8). Yet, medical school curricula often emphasize the teaching of medical facts and procedures rather than the learning of “doctoring” and how to communicate effectively with patients (9–11). Furthermore, the “hidden curriculum” of medical schools often promotes a dehumanizing view of patients and a value system that favors technical prowess, speed, and efficiency over interpersonal skills (12). When medical schools do incorporate training about communication and patient interaction into their preclinical curriculum, these skills can sometimes be de-valued, either implicitly or explicitly, in the clinical years (13). Decreasing empathy and compassion across the years of medical school and higher rates of burnout suggest that current curricula may actually erode students’ passion for helping others instead of building upon those values to educate physicians who compassionately provide cutting-edge care (14,15).

Existing strategies for boosting compassion in medical encounters tend to emphasize either the performative aspect or the affective aspect (4,16,17). Contemplative or meditation-based trainings emphasize the internal experience of the provider, (18,19) whereas interventions such as the “40 seconds of compassion,” in which short and simple scripts are added mechanically to the beginning and ending of encounters (4,7) target performative compassion.

The T. Denny Sanford Institute for Empathy and Compassion (TDSIEC) seeks to support the design and implementation of evidence-based, high-quality, integrated compassion training. To this end, we are examining the curriculum at the University of California San Diego (UCSD) School of Medicine to better understand how values and skills related to empathy and compassion are learned, and possibly unlearned, by our students. Here, we examine how medical students reflected on lessons about empathy and compassion in patient care through focus groups with fourth-year medical students. The analysis highlights a division and perceived incompatibility between the affective and the performative dimensions of compassion. Focus group participants related lessons of both types but struggled to combine them into viable clinical practices.

## Methods

We held in-person focus groups with fourth-year medical students in February 2020. All medical students in the fourth-year cohort were invited to participate in lunchtime focus groups scheduled to follow required lectures on campus.

Focus groups lasted for 90 min and included 5 to 12 students. Participants shared basic demographics in a write-in (not multiple choice) format. Consent was collected verbally to protect anonymity. Groups were audio recorded and professionally transcribed. Transcripts were reviewed by the study team to ensure accuracy of the transcription and to redact any personally identifiable information. The discussion guide focused on when, where, and how students learned to interact with patients and manage patient encounters (Appendix I). We focus this report on answers to the opening question: “What was the most memorable lesson you have learned about interacting with patients?”

To develop a set of “grounded” codes (20), 4 members of the research staff independently reviewed 2 transcripts, highlighting passages that were especially interesting. By examining these passages, we created a set of thematic codes and a set of context codes (Appendix II). Three independent coders completed the systematic coding of the corpus according to the codebook. Three of 6 transcripts were assigned to 2 coders who met to go over and come to a consensus on the coding. This *consensus coding* helped to maintain consistency among coders and over time (21) Because coders must come to an agreement in the process of consensus coding, we did not calculate intercoder reliability for these transcripts.

## Results

### Participants

From a cohort of 127 students, 48 fourth-year medical students participated in 6 in-person focus groups. Table 1 compares the demographic characteristics of the focus group participants with data on the full cohort collected by the medical school (including focus group participants). The focus group sample was broadly similar in demographics to the entire cohort. Participants indicated 15 different intended medical specialties, half of which were specialties related to primary care (n = 24). Participants were asked to self-describe their race or ethnicity and supplied 27 distinct descriptions.

**Table 1.** Demographic Characteristics of Focus Group Participants and Their Medical School Cohort.

Demographic Trait	Study Participants (n = 48)	Full Fourth-Year Cohort (n = 127)
**Gender**	60.4% (29) Female/ 39.5% (19) Male	52% Female/ 48% Male
**Mean age**	27 years	28 years
**Age Range**	24–34 years	24–45 years
**Top 4 Intended Specialties**	Internal Med., 25% (12) Emergency Med., 12.5% (6) Anesthesiology, 10.4% (5) Pediatrics, 10.4% (5)	Internal Med., 21.3% Emergency Med., 11.8% Psychiatry, 11.8% Pediatrics, 8.7%
**First in Family to Med School**	62.5% (30)	87.4%^a^
**Race or Ethnicity**		
“White” or “Caucasian”	35.4% (17)	40.9%
“Asian,” “Chinese,” or “Korean”	29.2% (14)	25.1%^b^
“South Asian” or “Indian”	14.6% (7)	13.4%^c^
“Hispanic” or “Mexican American”	6.3% (3)	7.1%
Unique Descriptions	14.6% (7)^d^	--

^a^ In medical school data, the question was “Did either of your parents go to medical school?” and the proportion who responded “no” is given.

^b^ Labeled as “Asian Chinese,” “Asian Japanese,” “Asian Korean,” “Asian Vietnamese,” or “Asian Other” in medical school data.

^c^ Labeled as “Asian Indian” in medical school data.

^d^ Includes Black, Arab, or mixed ancestry; comparable data not are calculable for the full cohort.

### Curricular Context

The curriculum at the UCSD School of Medicine integrates lessons about physician–-patient interactions throughout the course of study. Key sources for these lessons are the set of required “Clinical Foundations” courses, electives, and volunteer opportunities in the Student-Run Free Clinic (SRFC) (22–25). During the third year, clerkships provide hands-on learning about interactions with patients, and for this sample of fourth years, 1 clerkship (Pediatrics) ran a Master Clinician Program (MCP) with designated faculty giving specific feedback about compassionate care, patient interactions, and interpersonal skills.

### Types of Lessons

In response to the question, “What was the most memorable lesson you have learned about interacting with patients?” participants related specific lessons that fell into three main categories: (1) physical navigation of objects and bodies; (2) navigation of interpersonal interactions; and (3) perspective taking. Table 2 presents these lesson types with illustrative quotes from focus group participants (Table 2).

**Table 2.** Summary of Types of Lessons About Interacting With Patients Shared by Focus Group Participants.

Lesson Type	Definition	Example
**Physical Navigation**	Managing objects and bodies (sitting/standing, touch, draping, specula positioning, computer terminal location) Narrating actions	*I remember it was like learning how to do a cardiac exam where you have to ask the female to lift your left breast so I can listen underneath it. (FG1)* *[Gynecological teaching associate is] somebody who is showing you their body and allowing you to exam [sic] them in a way that they show you how to do it without being intrusive or offensive… a big part of the session [was] too like, “Oh, you should let me know before you touch me, at the knee and then at the thigh.” (FG4)*
**Interpersonal Navigation**	How to run a visit (timing, history taking, shared medical decision making) How to speak and relate to patients (managing emotions, approaching patients, accessible language)	*Like, you should never say, “I know how you’re feeling.” You should say, “I can imagine how you’re feeling.” It's a little thing, but I probably would have never thought that that's a bad thing to say before med school or if I wasn't told that. (FG2)* *So, you’re having to translate these things that were very much medical terminology into just simple everyday language and make sure that you don't say something that scares the patient or the family. (FG3)*
**Perspective Taking**	Understanding patient's experience and social determinants of health Acknowledging personal/cognitive limitations (bias, responses to time pressure)	*We are all guilty of it because we see a homeless person come in to ten different emergency departments asking for opioids and we all think the same thing. But we have to realize [that] we’re trained to, almost obsessively, recognize patterns. That's how we don't miss bad diagnoses or really red-flag things. (FG5)* *On a couple blocks they’ll bring in a patient with an exemplary disease from that block. […] I was definitely in tears after the guy with depression was just telling us what his life was like and how hard it's been for him.” (FG4)*

### Physical Navigation

Lessons about the physical navigation of objects and bodies included topics such as when to sit or stand during a patient encounter, how to move tools and drape blankets, how to touch a patient, and narrating actions to orient patients to the order and purpose of the exam. Though less common than other types of lessons discussed, these were the most concrete and detailed examples of what medical students learn about managing patient interactions. Some of the students who discussed these lessons clearly connected these highly specific actions to how patient experience can be centered and honored in the course of an encounter. Within comments about physical navigation, draping was a common example. Students emphasized the importance of being aware and conscientious about covering patients’ bodies with care. Another example, discussed in one group, was gynecological teaching associates—trained volunteers who guide students through the process of a pelvic exam while simultaneously being the subject of the exam (16) Students described simultaneously learning the technical skills of performing an exam and receiving feedback directly from a “patient” about how to make this potentially awkward experience more physically and interpersonally comfortable.

### Interpersonal Navigation

Interpersonal navigation focused on details pertaining to verbal communication. These lessons included managing the timing of a visit and taking histories, as well as lessons about how to speak to and relate to patients. For example, one participant reflected on how they have come to think of a successful patient encounter in relation to formalized checklists. Some students discussed the challenge of communicating complex medical information to people they did not know well. Another participant described becoming aware of how specific word choices and phrasing can make a difference in conveying empathy. Similarly, students discussed learning to use accessible, colloquial language with patients, even as they were learning to use medical jargon with their instructors and peers. Many participants discussed learning how to manage particularly emotional moments, describing specific experiences that clarified the importance of these techniques, such as preclinical sessions devoted to “breaking bad news.” Some students discussed the importance of reading nonverbal cues and how this awareness is an important aspect of patient-centered care.

### Perspective Taking

The third type of lesson was broadly related to perspective taking, where students gained awareness of patients’ life experiences and of students’ own biases and blind spots. These lessons were often connected with patient stories, either witnessed firsthand in clinical settings, or heard secondhand from mentors, peers, and the patients who visited their courses. Participants shared stories with happy or satisfying outcomes resulting from the diligence of conscientious practitioners. They also shared stories of mistakes and missed opportunities. Each of these stories pointed to the importance of seeing the patient as a whole person.

Perspective taking not only encompassed understanding the perspectives of patients but also becoming aware of one's own perspective. Participants discussed noticing their own biases and how they were affected by the medical setting they were learning to work in. Many students discussed the importance of treating the entire person, not just for more compassionate care, but also for better medical decision making.

### “Authenticity” and Time Pressure

Though many students noted the importance of physical and interpersonal navigation, we also heard skepticism about the utility of explicitly teaching these skills, often citing a difference between simulated lessons and “real life” with respect to authenticity and emotional impact. Some students appeared to evaluate lessons of interpersonal navigation according to their own affective response. This was common during discussions of sessions with simulated patients, for example, “It doesn't affect us as much because they’re not real patients and we kind of know that they’re actors.” (FG4) This comment subtly disparaged the lessons learned in simulated environments because of their muted emotional impact compared to working with “real patients.”

Similarly, other students emphasized innate or natural social skills over techniques taught in medical school. For example, one student contrasted explicit navigation lessons with watching a role model in action:

I feel so much of our teaching around empathy is so granular? […] A really big lesson for me was seeing a facilitator who was like, ‘Okay I’m going to pick up on what this person needs and support them’ and I think that's probably been the most helpful. Learning that you can trust your own social instincts and skills to pick up on what a patient needs instead of following a specific formula that we’re taught or specific acronyms that we’re taught. That just doesn't feel the most natural to me. (FG6)

In drawing this comparison, the student privileges the “natural” feeling associated with “social instincts” over the “granular” lessons about empathy often offered in the preclinical courses. Though this participant did also acknowledge the utility of the stepwise approach taught in medical school, the ultimate implication is that students already naturally know how to discover what patients need. In a similar comment, another student described frustration with the stiff and structured evaluation of interpersonal skills:

I felt, in some ways the checklists and things, it was really good to come back to this foundation but it's like patient dignity, draping, stuff like that. But, like, in other ways, it was hindering in a way because I’m not going to be knocked on this in real life. I’m just going to treat my patients like they are a person. (FG1)

Once again, this comment implies that compassionate care is a simple matter of treating everyone “like they are a person.” Though acknowledging the utility of “foundational” checklists, this student's attitude appears to justify the dismissal of checklists in “real life.”

The assumption that empathy is natural can also imply that it need not or cannot be taught. This is illustrated by one participant's reflection on navigating an encounter with a patient who presented pressing emotional needs.

They came in for one problem and then ended up having this really traumatic episode that they wanted to talk about. And it's not like I was going through the checklist […] It was just like, oh, this person is going through this terrible thing. Let me just help them out as best as I can because I’m a person […] I feel like once you do that empathetic part, then everything else falls in line. (FG3)

In this account, helping the patient becomes something simple (“let me *just* help them” [emphasis added]) and good doctoring is described as flowing naturally from being an empathetic person rather than from rigorous professional training.

Alongside comments about natural and authentic compassionate care, participants also described their struggles to live up to these ideals under the pressures of hospital and clinical environments. One participant reflected, “I’ve assumed that these residents and attendings lack the empathy, but it's because the system has driven it out of them[…]. And it's unfortunate because then we mirror that.” (FG5) Many students also described empathy and efficiency as a tradeoff that must be made in most medical environments. As one student put it, “You have 15 min to see a patient or less, and how do you maintain your humanity while having to do ‘x’ amount of work?” (FG3) Another commented, “In medicine in general right now, the priorities are with efficiency, and physicians are not given the time that they’d like to be able to foster these relationships, and really sit down and dig through charts to find out, like why is this patient in so much pain?” (FG5)

This prioritization of efficiency over empathy and compassion affected some students quite deeply. One student described how “It's been drilled into me that efficiency is so much more important than being empathetic.” This student explained, “If my patient was really sad I felt like I would try to comfort them, but [thought], ‘I have to see three other patients, or else I’m going to get yelled at by my intern or resident.’” (FG6) According to this account, compassion was undermined by aggressively delivered negative feedback focused on efficiency which created an internal conflict for this student and disrupted attention to the emotional needs of patients. The concern that empathic and compassionate care would be undermined by the medical environment in the name of efficiency was echoed by many students across all focus groups.

## Discussion

The three categories of lessons identified here (physical navigation, interpersonal navigation, and perspective taking) reflect the division between performative versus affective compassion. Where perspective-taking lessons target the affective aspect of empathy and compassion, the more explicit physical and interpersonal navigation lessons target the performative component. Many of the students we spoke to struggled to connect the performative techniques of physical and interpersonal navigation with the broader emotional work of perspective taking in practice. Some comments described navigation techniques as forced, unnatural, or inauthentic. Other comments expressed the notion that “true” empathy was an instinct or natural social ability, implying that it could not be taught and could only be achieved through natural, unscripted connection with each patient. At the same time, students expressed concern about not having enough time to be authentically empathic as they start to take on more clinical responsibilities.

Together, these attitudes suggest an overidentification of compassion with perspective taking and too little appreciation for the small ways that physicians perform their respect for a patient's humanity. While students generally described perspective-taking lessons as more valuable and gratifying, accounts of physical and interpersonal navigation illustrate that many do come to rely on the specific techniques regarding, for example, draping or “breaking bad news.” Ideally, perspective taking motivates these techniques by teaching students to recognize that they cannot know everything about their patients and gives meaning to the small actions that convey compassion and respect, regardless of how well they know a patient. However, some students appear to harbor the unattainable expectation that authentic compassionate care must flow naturally from deep individual connection and understanding between physicians and patients. No wonder, then, that combining efficiency with compassion is viewed as extremely difficult, if not impossible.

Given what we know from counseling and studies of implicit bias, the notion that acting with empathy and compassion is innate or natural rather than a skill to be developed is particularly worrisome among elite preprofessionals. In other professional training, such as psychological counseling, the technical aspects of interpersonal communication receive special attention because practitioners acknowledge that instinctive reactions can often perpetuate problematic behavior (26–28). Furthermore, unexamined interpersonal interactions may reflect and perpetuate implicit biases (29–31). Entire courses focus on improving microskills such as listening, being present, nonverbal communication, open-ended questions, alliance building, goal setting, and reflecting feeling, content, and meaning; competence in these domains is not assumed. Other focused perspective-taking skills and self-awareness are also key elements of many counseling and clinical psychology curricula.

### Limitations

The focus group participants quoted here were a self-selected group of students in a single medical school with its own unique history and curriculum. While we did not ask specifically about empathic or compassionate interactions, that frame may have been implicit in the sponsorship of the study by the TDSIEC. Future work might look at the most memorable lessons that emerge from other schools’ curricula or explore how physical and interpersonal navigation techniques may be better connected to contemplative strategies.

## Conclusion and Ongoing Work

Many of the medical students who participated in this study have developed an appreciation for the building blocks of compassionate care but more can be done to clarify how perspective taking and self-reflection complement concrete navigation techniques. Some students and instructors may need only to read this analysis to appreciate this tacit connection. However, creating a culture where the many facets of compassionate care are regarded as equally important and necessary in the practice of medicine will require more intentional and sustained effort.

Students may benefit from more explicit guidance on the relationship between the perspective taking and navigation techniques already present in the undergraduate medical curriculum. As this analysis suggests, perspective-taking exercises should be introduced as motivating the need to utilize best practices in navigating interactions, rather than as an end in itself. Lectures, workshops, and course materials could be developed to enhance faculty and student understanding of how physical and interpersonal navigation—as well as perspective taking—contribute to compassionate care. Training faculty in compassionate communication will also help them to nurture these skills in the context of clinical interaction. Finally, assessment and evaluation measures could be updated to provide students with more substantive feedback regarding empathy and compassion practices. For example, simulated patient experiences can be developed to reflect the time constraints physicians are increasingly facing and offer strategies for practicing compassion under time constraints.

Our findings also point to an opportunity for medical education to learn from other caring professions. While behavioral scientists and psychologists teach clinical foundations at the UCSD, the curriculum is set by the school of medicine. Future research could examine the differences between the curricular approaches in medical training and clinical psychology training. For example, a comparison between different professional approaches to managing psychological trauma might uncover specific techniques, already well-established in the behavioral sciences, which could better prepare medical students for compassionate interactions with patients.

We intend to use the insights described here to strengthen and clarify the existing commitment to compassion in healthcare and develop techniques that can be used at other schools of medicine. We have begun to develop new approaches to training faculty, for example, through the expansion of the existing MCP across all clerkships. The goal is to provide individualized medical student mentorship wherein compassionate care may be modeled in a real-life setting.

We are hopeful that these initiatives will contribute to a culture that honors the complementarity of both navigation and perspective-taking practices. By bringing the two aspects of compassionate care into clearer dialogue, perhaps more physicians can learn to identify their small acts of compassion as worthy of that label and appreciate how crucial those acts are, especially when time is short.

## Appendices

### Appendix I: Discussion Guide for Focus Groups With Fourth-Year Medical Students

**Introduction**

[Facilitator personal introduction.]

Thank you for joining us to discuss your experience of the medical curriculum here at UCSD. I am here to learn about your experiences in medical school, specifically, how you learned to manage interactions with patients.

Our conversation will be recorded, but your comments will be kept anonymous. We will not include your name or identifying information in any of our reports or publications. You probably know each other, so I ask you to be respectful of each other's privacy. While it is fine to tell other people about the topics we discuss here, please do not repeat individual comments to others. I also request that you try not to interrupt or talk over each other or have side conversations.

My job is to maintain a safe space, help everyone to contribute to the conversation, and keep you on topic. However, I am just as interested in hearing what you have to say to each other as I am in hearing what you have to say to me. I encourage you to respond to and discuss these topics as you might at a dinner party.

Please take a minute to look over and sign the consent forms. [Review major points of consent form.]

[Distribute demographic form.] Please take a moment to fill out this quick questionnaire to help us report who made up this group.

[Begin recording.]

**I. Assent**

I have X participants. As we go around the room, please acknowledge that we are recording this session by stating, “I agree” to the following question:

Do you agree to participate in this focus group?

**II. Learning to interact**

Please take a moment to think about how you have learned to manage your interactions with patients during your medical education so far. Specifically, **what was the most memorable lesson you have learned about interacting with patients?** Before we discuss, I invite you to jot down a few thoughts.

I’d like to hear from everyone about a memorable lesson. Would someone like to start us off?

- Does anyone have a similar story to share?
- [write down the lessons or themes]
- Thinking about your time as a medical student, do you feel that you have gotten enough of these lessons? Too many? Just right?

When you were taught to conduct a physical exam, how were you instructed to interact with the patient?

How have you learned to present test results to patients?

Would anyone be willing to share a story about **a time you were surprised by a patient**?

- How did you handle this? Did you get guidance from your supervisor/attending/resident?
- Does anyone else have a similar experience to share?

**III. Interacting with specific types of patients**

Do you feel your training has focused on a **particular type of patient**?

For example, do you feel prepared to interact with patients who have **low health literacy**?

What about patients who are **not cooperative**?

What about very **knowledgeable patients**?

Has anyone had any **experience with patients bringing in their information**?

**IV. Learning Epic or EHR**

Tell me about **how you have learned to manage the electronic medical record** while interacting with patients.

- What has been most helpful?
- What has been most frustrating?

**V. Closing Questions**

Has it been difficult to maintain your compassion as training has gone on?

What do you think will be the biggest change you will have to deal with in your careers?

- As medical students, how have you been prepared to deal with this change?

### Appendix II: Full Codebook

**Table:**

Grouping	Label	Definition	Coding Rules
**Themes**			
	Time	Discussions of time management, efficiency, time constraints, etc.	Code entire story
	Grades	Discussion of evaluation, testing, grades, etc.	Code entire story
	Variation	Narratives that suggest inconsistencies or variations across contexts (such as between preclinical and clinical; between rotations/attendings; between simulation and reality); includes differences in expectations	Code entire story
	MD Identity	Discussions of feeling like a doctor, what it means to be a doctor, or what kind of doctor someone wants to be. Also include discussions of choosing specialities and discussions of developing one's own clinical style.	Code entire story
	Emotions	Discussions of managing, coping with, expressing, understanding, coming to terms with emotions (of self or other). Include discussion of debriefing and vulnerability; Include discussions of *experiencing* empathy or compassion	Code entire story
	Humanity	Discussions of seeing the patient as a person, understanding their background or social history, and any mentions of the concept of humanity. Include discussions of *witnessing* empathy or compassion, or lack thereof.	Code entire story
	Tech Topics	Topics of interest to the Center for Technology and Empathy: Discussions of and answers to questions about medical technologies (include patient generated data AND hyperliterate patients, genomics, AI, telephone translators, and telemedicine)	Code entire section of focus group discussion devoted to these topics
**Contexts**			
	Observations	Discussion of lessons or experiences in the clinic that are learned through watching others	Code whole story
	Coaching	Discussions of lessons learned in clinical work but through direct coaching from another person (i.e., walking through epic templates or deliberate debriefing)	Code whole story
	Lecture	Discussions of lessons learned in lectures or didactic formats	Code whole story
	PBL	"Practice based learning”—Discussions of lessons learned through interactive learning, including role playing, simulated patient work, and visits from patient representatives	Code whole story
	Specialties	All specific mentions of specialties	Code only the word
**Lessons**			
	Interaction Techniques	Discussions of specific techniques for managing patient encounters including how to talk to a patient, make eye contact, timing, etc. Also include managing objects in the room such as drapes, speculums, computer screens/terminals, etc.	Code specific technique in the shortest number of words possible, ideally a sentence
	Lessons—Individual	Lessons or strategies that individuals may apply when facing challenges in practice	Code specific lesson in the shortest number of words possible, ideally a sentence
	Lessons—Institutional	Ideas for how UCSD SoM can improve training for students (include reports of other institutions)	Code specific idea in the shortest number of words possible, ideally a sentence

## Key Messages

- Students remember and integrate lessons about both performative and emotional dimensions of compassion.
- Students seek practical strategies for applying perspective taking in clinical practice.
- Students may perceive compassion and efficiency as mutually exclusive.
